# Biosynthesis, Engineering, and Delivery of Selenoproteins

**DOI:** 10.3390/ijms25010223

**Published:** 2023-12-22

**Authors:** David E. Wright, Patrick O’Donoghue

**Affiliations:** 1 Department of Biochemistry, The University of Western Ontario, London, ON N6A 5C1, Canada; dwrigh56@uwo.ca; 2 Department of Chemistry, The University of Western Ontario, London, ON N6A 5C1, Canada

**Keywords:** amber codon, aminoacyl-tRNA synthetases, elongation factor, genetic code expansion, opal codon, selenocysteine, selenocysteine insertion sequence (SECIS), selenoproteins

## Abstract

Selenocysteine (Sec) was discovered as the 21st genetically encoded amino acid. In nature, site-directed incorporation of Sec into proteins requires specialized biosynthesis and recoding machinery that evolved distinctly in bacteria compared to archaea and eukaryotes. Many organisms, including higher plants and most fungi, lack the Sec-decoding trait. We review the discovery of Sec and its role in redox enzymes that are essential to human health and important targets in disease. We highlight recent genetic code expansion efforts to engineer site-directed incorporation of Sec in bacteria and yeast. We also review methods to produce selenoproteins with 21 or more amino acids and approaches to delivering recombinant selenoproteins to mammalian cells as new applications for selenoproteins in synthetic biology.

## 1. Discovery of the Genetic Code and the Mechanism of Protein Synthesis

Following the discovery of the structure of deoxyribonucleic acid (DNA), Francis Crick proposed the central dogma of molecular biology that describes how the genetic information in cells flows from DNA and RNA to proteins [1]. Crick’s sequence hypothesis envisioned that the sequence of nucleic acids corresponds to the amino acid sequence of proteins and is used as a template for amino insertion during protein synthesis [1]. Indeed, in all cells, the DNA of a protein-coding gene is converted into ribonucleic acid (RNA) known as messenger RNA (mRNA) through a process called transcription. The resulting mRNAs are used by the ribosome as templates to direct the accurate insertion of each amino acid during protein synthesis [2]. The ribosome is a large ribonucleoprotein particle consisting of large, small, and 5S ribosomal RNA (rRNA) subunits as well as many tightly bound ribosomal proteins. The ribosome is an RNA enzyme (ribozyme) that uses its RNA components to catalyze the peptide bond reactions that link each successive amino acid to the next in the growing polypeptide chain [3]. Each trinucleotide sequence (codon) in the mRNA is read by the ribosome according to the genetic code table, which was deciphered by Nirenberg [4] and Khorana [5] in 1965.

**Figure 1:**
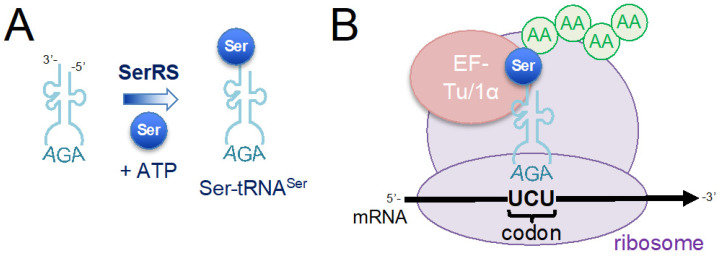
Schematic of protein synthesis by messenger RNA (mRNA) translation at the ribosome. The mRNA is translated into proteins at the ribosome, which catalyzes the formation of peptide bonds between successive amino acids (AAs). Amino acids are brought to the ribosome by aminoacylated tRNAs (aa-tRNAs) and the protein sequence is determined by the tRNA anticodon binding to complementary trinucleotide sequences in the mRNA, which are called codons. The aminoacyl-tRNA synthetases (aaRSs) are responsible for recognizing a cognate tRNA and the corresponding amino acid. (**A**) For example, seryl-tRNA synthetase (SerRS) catalyzes a two-step reaction in which serine (Ser) and adenosine triphosphate (ATP) are used to form a seryl-adenylate intermediate, which is subsequently used to ligate the Ser moiety onto tRNA^Ser^. SerRS recognizes tRNA^Ser^, shown with an AGA anticodon that decodes the UCU codon for Ser. To clearly depict the codon:anticodon duplex, the tRNA is shown in the 3′ to 5′ orientation, and the mRNA is shown in the normal 5′ to 3′ orientation. (**B**) Following aminoacylation, aa-tRNAs are each bound by elongation factor thermal unstable (EF-Tu) in bacteria or EF-1α in archaea and eukaryotes. During translation elongation, the complex of elongation factor and aa-tRNA is delivered to the ribosome, where correct pairing between codon and anticodon leads to the incorporation of the amino acid in the growing polypeptide chain.

The genetic code consists of 64 codons, 61 of which correspond to one of the 20 standard proteinogenic amino acids. Three codons (UAG, UGA, and UAA) are termination signals, marking the end of the protein sequence [6]. Adapter molecules called transfer RNAs (tRNAs) bring amino acids to the ribosome and determine the amino acid sequence of the polypeptide chain by binding to a specific codon or set of codons in the mRNA with a complementary tRNA anticodon [7] (Figure 1). Aminoacyl-tRNA synthetases (aaRSs) catalyze the ligation of amino acids to their cognate tRNAs through an adenosine triphosphate (ATP)-dependent reaction, producing aminoacyl-tRNA substrates for protein synthesis [8] (Figure 1).

Accurate translation of the genetic code requires that tRNAs are aminoacylated by a specific aaRS with the correct or cognate amino acid that corresponds to the codon(s) read by that tRNA’s anticodon [8]. Amino acid substrate selectivity is determined by the structure and chemical environment of the aaRS active site. Some aaRSs possess editing activities to hydrolyze tRNAs that are mis-aminoacylated with a chemically similar but non-cognate amino acid [9]. The aaRSs must also discriminate their cognate tRNA from the large pool of structurally similar tRNA molecules through recognition of a key set of identity element nucleotides [10,11]. Together, the selectivity of each aaRS for the cognate amino acid and tRNA ensures accurate ligation of the cellular tRNA pool and faithful translation of the genetic code [10].

## 2. A Natural Expansion of the Genetic Code with Selenocysteine

**Figure 2:**
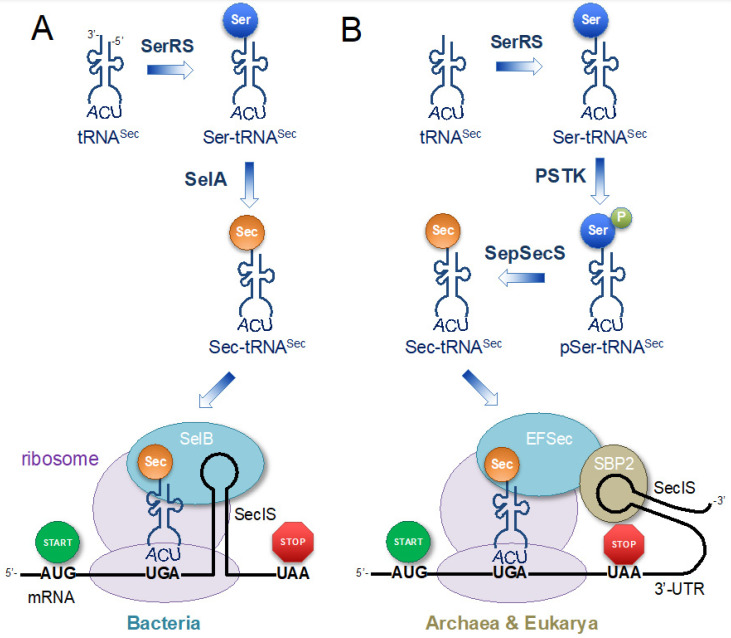
Translation with selenocysteine (Sec) in nature. The tRNA^Sec^ is aminoacylated with serine (Ser) by seryl-tRNA synthetase (SerRS). (**A**) In bacteria, selenocysteine synthase (SelA) converts Ser to Sec on tRNA^Sec^, while in (B) archaea and eukaryotes, Ser-tRNA^Sec^ is phosphorylated by phosphoseryl-tRNA^Sec^ kinase (PSTK), followed by conversion to Sec by Sep-tRNA:Sec-tRNA synthetase (SepSecS). Sec-tRNA^Sec^ is localized at the UGA codon by a specialized elongation factor that binds an RNA hairpin loop, the selenocysteine insertion sequence (SECIS) that occurs downstream of the Sec (UGA) codon. (**A**) In bacteria, SECIS is present directly downstream of UGA in the open reading frame (ORF) and the tRNA^Sec^-specific elongation factor (SelB) binds Sec-tRNA^Sec^ and localizes it at the UGA recoding site by also binding to SECIS. (**B**) In archaea and eukaryotes, SECIS is present in the 3′ untranslated region (UTR); SBP2 binds SECIS and to the elongation factor (EFSec), which localizes Sec-tRNA^Sec^ at the UGA recoding site.

The genetic code was originally thought to be universal in all species; however, diverse exceptions to the universal genetic code have emerged [12]. While there are examples of codons taking on different meanings (codon reassignment), the standard set of 20 different kinds of proteinogenic amino acids was also viewed as immutable. In the late 1980s, the first exception was discovered in some UGA termination codons that directed the site-specific and co-translational incorporation of the 21st amino acid, selenocysteine (Sec). The Sec-decoding trait was subsequently found only in a few select proteins in some species representing all three domains of life [13,14,15] (Figure 2).

Two partially conserved yet distinct mechanisms for Sec incorporation into proteins evolved in bacteria as compared to archaea and eukaryotes (Figure 2). In both mechanisms, the meaning of selected UGA stop codons is converted from a stop to a sense codon that encodes Sec [15,16,17]. In all Sec-decoding organisms, Sec is synthesized from serine (Ser) on its cognate tRNA (tRNA^Sec^). First, tRNA^Sec^ is aminoacylated by an endogenous and normal seryl-tRNA synthetase (SerRS) to produce the intermediate, Ser-tRNA^Sec^ (Figure 2) [18,19]. The same SerRS also aminoacylates tRNA^Ser^, and both tRNA^Ser^ and tRNA^Sec^ share structural features, including a large extra variable arm. Another conserved element is the selenophosphate synthetase (SelD or selenophosphate synthetase 2, SPS2) that provides an activated and phosphorylated form of Se, and an essential substrate needed to convert Ser-tRNA^Sec^ to Sec-tRNA^Sec^ [20]. Beyond this point, the mechanism in bacteria is divergent compared to eukaryotes and archaea. The bacterial selenocysteine synthase (SelA) directly converts Ser-tRNA^Sec^ to Sec-tRNA^Sec^ [21], while in eukaryotes and archaea the Ser-tRNA^Sec^ is first phosphorylated by *O*-phosphoseryl-tRNA^Sec^ kinase (PSTK) [22,23], followed by conversion of phosphoseryl-tRNA^Sec^ to Sec-tRNA^Sec^ by *O*-phosphoseryl-tRNA:selenocysteinyl-tRNA synthase (SepSecS) [24,25,26] (Figure 2). SepSecS and SelA are distantly related members of the pyridoxal phosphate (PLP)-dependent enzyme family [27]. 

Recoding to Sec relies on an RNA hairpin structure in the mRNA. The selenocysteine insertion sequence (SECIS) occurs downstream of the Sec-encoding UGA codon. In bacteria, SECIS is directly downstream of the UGA codon at a distance of 16–37 nucleotides between UGA and the apical loop of SECIS [28], which is often located within the open reading frame (ORF) of the selenoprotein gene (Figure 2). In archaea and eukaryotes, the SECIS is found more distantly downstream and is located in the 3′ untranslated region (UTR) of the selenoprotein genes [29] (Figure 2). Fascinatingly, in some species, UGA codons are not used to encode Sec, and instead, the SECIS element functions to recode other stop codons (UAG and UAA) and even 10 different sense codons to Sec, including the Cys UGU codon in *Aeromonas salmonicida*. These species also harbour a tRNA^Sec^ with the anticodon complementary to the sense codon that is designated for re-coding to Sec [30]. 

Sec-tRNA^Sec^ does not interact with the normal translation elongation factors, EF-thermal unstable (EF-Tu) in bacteria or EF-1α in archaea and eukaryotes. Instead, Sec incorporation relies on a specialized elongation factor that shares homology with EF-Tu and EF-1α. In bacteria, the SelB elongation factor [31] binds to both Sec-tRNA^Sec^ and the SECIS stem-loop to co-localize the Sec-tRNA with the adjacent UGA codon (Figure 2A). The eukaryotic elongation factor Sec (EFSec) [32], and the archaea SelB (aSelB) [33] fulfill a similar role in binding Sec-tRNA^Sec^; however, an additional protein binds the SECIS element. In eukaryotes, SECIS binding protein 2 (SBP2) interacts with both the SECIS and EFSec [34] to localize the distant SECIS element to the target site for recoding [32] (Figure 2B). In archaea, the aSelB protein binds tRNA^Sec^ [33] and positions it at the UGA codon by presumably interacting with an as-yet unidentified SECIS binding protein [35].

## 3. Distribution of Selenoproteins

Recoding of UGA to Sec occurs in all three domains of life, Bacteria, Archaea, and Eukarya, but not in all species. Only approximately 20% of bacteria [36,37,38] and 14% of archaea [37,38] contain the Sec-decoding trait and selenoproteins. In eukaryotes, the distribution and number of selenoprotein genes varies greatly. Selenoproteins are found in all vertebrates [39], while several species of insects lack selenoproteins [40]. Selenoproteins are absent from most fungi, with only nine species identified as containing selenoproteins [41]. The Sec-decoding trait was identified in several species of algae [42,43,44], but not in higher-order plants [44]. The Sec-decoding trait is conserved in all animals, including humans. The number of selenoproteins varies greatly among eukaryotic organisms. Mammals and aquatic invertebrates have relatively large selenoproteomes with 15–30 different selenoprotein genes, while other multicellular species, including flies, bees, and worms encode just one or only a few selenoproteins. Phylogenetic analysis suggests that the large selenoproteomes of aquatic invertebrates were lost in many terrestrial species due to the replacement of Sec with Cys [44]. 

## 4. Human Selenoproteins

**Figure 3:**
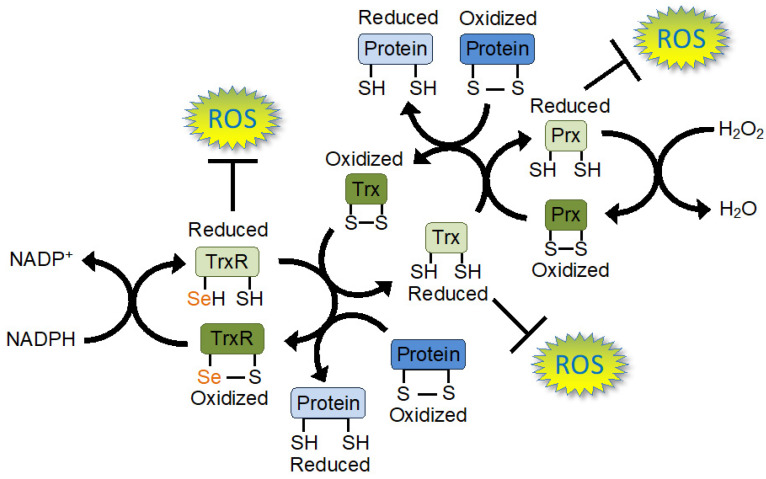
Electron flow through the thioredoxin (Trx) redox network. Electron flow through the Trx system is mediated by thioredoxin reductase (TrxR) using electrons from nicotinamide adenine dinucleotide phosphate (NADPH). TrxR reduces Trx before the oxidized TrxR is re-reduced using electrons from NADPH. Trx then reduces peroxiredoxin (Prx), or other target proteins, before oxidized Trx is reduced again by TrxR. Prx reduces reactive oxygen species (ROS) such as hydrogen peroxide (H_2_O_2_). TrxR and Trx can also directly reduce ROS, providing the cell with a powerful ROS defence system. TrxR and Trx also act on a wide variety of other proteins with diverse functions, and the Trx system regulates proteins involved in many cellular functions. TrxR contains a highly nucleophilic selenium (Se) atom in its active site in the form of selenocysteine (Sec). Sec endows TrxR with a catalytically efficient reductase activity that is important in defending the cell against oxidative stress and in redox signalling.

There are 25 selenoproteins encoded in the human genome [45]. While the function of most of these selenoproteins remains unknown, the majority of characterized and functionally defined selenoproteins have oxidoreductase activities [46]. The best-characterized selenoprotein families are the thioredoxin reductase (TrxR), the glutathione peroxidase (GPx), and the iodothyronine deiodinase (DIO) protein families, which all have important roles in redox metabolism [47]. Two major antioxidant systems in mammals are the Trx [48] and the glutathione (GSH) [49] systems, which protect cells against reactive oxygen species (ROS) and fulfill important cellular signalling functions. The Trx system is driven by three isoforms of the Sec-containing reductase, TrxR (Figure 3). The GSH system relies on the Sec-containing GPx enzymes. 

### 4.1. Glutathione Peroxidases 

There are eight GPx isoforms in human cells, and five (GPx1, GPx2, GPx3, GPx4, and GPx6) are selenoproteins with Sec in the catalytic active site. The three other isoforms use an active-site Cys in place of Sec [50]. GPxs, using GSH as a reductant and act to reduce H_2_O_2_ to water or organic hydroperoxides to alcohols [50]. GPx1, GPx2, GPx3, and GPx6 function as homotetramers, while GPx4 is a monomer [50]. GPx1 is the most abundant isoform and is ubiquitously expressed in both the cytoplasm and mitochondria. GPx1 together with GSH reduces H_2_O_2_ and low-molecular-weight hydroperoxides [51]. GPx2 is similar to GPx1 yet mainly expressed in the gastrointestinal system. GPx3 is secreted from cells and is found in human plasma as a glycosylated protein. GPx3 can also use Trx as a reductant in addition to the GSH monomer [50]. GPx4 is a membrane-associated protein, where it protects cell membranes from oxidative stress, and GPx4 is the only isoform that can reduce complex lipid hydroperoxides. GPx6 is not well characterized. The GPx6 isoform is expressed mainly in embryonic cells and in epithelial cells of the olfactory system where it may regulate the metabolism of odorants [51].

### 4.2. Thioredoxin Reductase, a Critical Selenoprotein in Redox Biology

Mammalian TrxR is a key redox regulator in mammalian cells and is a selenoprotein that is a powerful oxidoreductase containing selenium (Se) in the form of Sec [52]. TrxR, along with its major substrate, Trx, compose one of the major disulphide reduction systems in the cell (Figure 3) [53]. Sec is an analogue of Cys with Se taking the place of sulfur [54]. The Se in Sec produces a stronger nucleophile than Cys, making Sec-containing reductases more efficient electron donors for catalyzing redox reactions due to their lower redox potential [55]. Cys to Sec substitutions in Cys-containing reductases can indeed reduce redox potential and increase enzyme activity [55], while Sec to Cys substitutions in TrxR eliminate its activity with some substrates and drastically reduce its activity with others [56,57]. Due to its high nucleophilicity, Sec is also the target of clinically relevant TrxR inhibitors, providing a unique mechanism to specifically target the activity of this selenoprotein [58,59,60,61]. TrxR inhibitors include the platinum-containing compound, cisplatin, which is a widely used chemotherapeutic drug, and the gold-containing compound, auranofin, a treatment for rheumatoid arthritis [62] and a potential anti-cancer drug [63]. TrxR is the main driver of the Trx system (Figure 3), which is involved in oxidative stress responses in eukaryotes, bacteria, and archaea [64]. 

#### 4.2.1. Thioredoxin System in Cells

In addition to the glutathione system, the Trx system is one of the two main redox regulatory systems in mammalian cells [48] (Figure 3). In mammalian cells, there are three genes encoding three main TrxR isoforms, TrxR1, TrxR2, and TrxR3 [65]. TrxR1 localizes to the cytosol, TrxR2 localizes to the mitochondria, and TrxR3 is present primarily in the testes [65]. The Trx system consists of several enzymes that work together to transfer electrons from nicotinamide adenine dinucleotide phosphate (NADPH) to a wide range of substrates to maintain the redox balance of the cell, protect against oxidative damage caused by ROS, such as H_2_O_2_, and to control the function of various proteins through redox regulation (Figure 3) [48].

In the Trx system, TrxR transfers electrons from NADPH to Trx [66,67,68]. After being reduced by TrxR, Trx reduces cellular proteins, such as peroxiredoxin (Prx), before being recycled again by TrxR (Figure 3) [48]. Similarly, after receiving an electron from Trx, Prx can then reduce ROS such as H_2_O_2_ before being recycled again by Trx [69] (Figure 3). Prx can reduce several different ROS [70], including H_2_O_2_ [71], peroxynitrate [72,73], and alkyl hydroperoxide [74]. Some ROS can also be directly reduced by TrxR (lipid hydroperoxides [75] and H_2_O_2_ [56]) and Trx (hydroxyl radicals [76]), providing multiple layers of oxidative defence (Figure 3). 

TrxR also acts on several other oxidized proteins besides Trx [68], and Trx also functions to reduce many proteins beyond Prx (Figure 3) [77]. In addition to oxidative defence, the Trx system regulates many cellular processes including gene expression, embryonic development, cell proliferation, and apoptosis [78]. The Trx system is involved in diverse processes because Trx-dependent redox activities regulate a wide range of protein substrates, including Prx, ribonuclease reductase, phosphatase and tensin homolog, and transcription factors, such as NF-κB, AP-1, and the glucocorticoid receptor [79,80]. 

#### 4.2.2. Thioredoxin System in Disease

Because the flow of electrons into the Trx system depends on TrxR activity, and because of the diverse roles of the Trx system, TrxR is involved in the development and progression of human diseases [77,81,82,83]. Dysregulation of the Trx system has been observed in many diseases, such as Alzheimer’s disease [84], rheumatoid arthritis [85], asthma [86], various forms of cardiovascular disease [87], and other disorders [77,81,82,83]. TrxR1 is involved in several types of cancer [88], including non-small-cell lung carcinoma [89], renal cell carcinoma [90], thyroid cancer [91], breast cancer [91], cervical carcinoma [92], and colorectal cancer [93]. Overactive TrxR activity is linked to the chemotherapeutic resistance of some cancer cells by helping to defend tumours against ROS generated by radiation-based chemotherapies [94]. The activity of TrxR is also used as a diagnostic maker for the early detection of lung [95] and breast cancers [96]. Anti-cancer drugs, such as ethaselen that targets TrxR activity, were developed to combat drug-resistant lung cancers [97].

### 4.3. Diverse and Unknown Functions of Human Selenoproteins

The thyroid hormone is regulated locally at the tissue level by DIOs, which convert the prohormone thyroxine (T4) to its active form (triiodothyronine, T3) by 5′-deiodination, or convert T4 and T3 to inactive forms by 5-deiodination [98]. There are three Sec-containing DIOs in humans, which vary in where they are expressed, and which reactions they catalyze [99]. DIO2 activates the thyroid hormone by converting T4 to T3 by a 5′-deiodination reaction, while DIO3 deactivates the thyroid hormone by converting T4 and T3 to relatively inactive lesser iodothyronines (reverse triiodothyronine, rT3, and 3,3′-diiodothyronine, T2) by a 5-deiodination reaction [100]. DIO1 can catalyze both 5′- and 5-deiodination, activating or deactivating the thyroid hormone, respectively [100]. DIO1 is found primarily in the liver, kidney, and thyroid, and in many other tissues at a lower level in adult mammals. DIO2 is found primarily in the uterus, brown adipose tissue, central nervous system, pituitary gland, and placenta, while DIO3 is found primarily in the brain, ovary, placenta, pregnant uterus, testis, and the skin [101]. DIOs are involved in a wide range of processes during development and in adults [102] and play a major role in differentiation during development [103]. For example, DIO2 and DIO3 are required for the development of the cochlea. DIO3 knockout mice have premature cochlear differentiation [104], while DIO2 knockout mice have retarded cochlear development [105], both of which result in deafness [104,105]. 

SPS2 is also a Sec-containing selenoprotein in humans [106], and is involved in the production of selenophosphate from selenide, which is required for the conversion of Ser-tRNA^Sec^ to Sec-tRNA^Sec^ [107]. Selenoprotein P is a unique selenoprotein. While most mammalian selenoproteins have one SECIS in the 3′-UTR, human selenoprotein P has two SECIS elements, and contains 10 Sec residues [108]. Selenoprotein P functions to transport Se from the liver to different cells around the body and to reduce phospholipid hydroperoxides [108]. Selenoprotein R, which is also known as methionine-R-sulfoxide reductase B1 (MsrB1), catalyzes the reduction of oxidized methionine residues to repair protein damage due to oxidative stress [46]. 

The function of other human selenoproteins remains uncharacterized or only partially understood [109]. Selenoprotein O occurs in the mitochondria and is proposed to have a kinase function [110], but this has not yet been confirmed experimentally. Selenoproteins T, W, H, and V have a redox motif, suggesting a potential redox function [111]. A version of selenoprotein I terminated at the UGA Sec codon, and lacking Sec, functions as an ethanolime phosphotransferase [112], but the function of full-length, Sec-containing selenoprotein I has not yet been determined [53,109]. 

Selenoprotein K and selenoprotein S are both endoplasmic reticulum (ER) resident proteins and associated with complexes involved in the ER-associated degradation of misfolded proteins, suggesting a possible role in protein homeostasis and quality control [53,113,114]. The function of selenoprotein F is still unclear, but it has been implicated in protein folding and secretion in the ER [115]. Selenoprotein M is another ER-resident protein that is expressed in the brain and was implicated in calcium release from the ER [109,116], but its specific role is not clear. Selenoprotein N is also found in the ER and includes a calcium-binding domain and a separate domain with an unknown function that contains Sec [109]. 

## 5. Engineered Genetic Code Expansion Systems for Sec and Beyond

### 5.1. Pyrrolysine: Another Natural Expansion of the Genetic Code

In the early 2000s, the UAG termination codon was found to be decoded as an unusual lysine derivative, pyrrolysine (Pyl), in the methanogenic archaeon *Methanosarcina barkeri* [117]. Pyl was found in the active site of three archaeal methyltransferases [118] in several species of the *Methanosarcinaceae* family and the Pyl residue is crucial for utilizing methylamines as a carbon source [119]. Pyl is incorporated into proteins by reassignment, rather than recoding, of the UAG codon. In contrast to Sec, where only selected codons are designated for re-coding to Sec, the Pyl system re-assigns all instances of the UAG codon from stop to Pyl [120]. Like Sec, Pyl is also a natural expansion of the genetic code. The Pyl system requires the pyrrolysyl-tRNA synthetase (PylRS) and a cognate tRNA^Pyl^ that includes a CUA anticodon to decode UAG codons. The tRNA^Pyl^ is aminoacylated with free Pyl through an ATP-dependent reaction [121,122]. Pyl-decoding organisms also biosynthesize Pyl from two lysine residues with the activity of three genes: *pylB*, *pylC*, and *pylD* [123]. 

### 5.2. Engineering Genetic Code Expansion

The discovery of exceptions to the genetic code in nature, such as recoding of selected UGA codons to Sec, or reassignment of UAG to Pyl, provided both the inspiration and the molecular machinery required for a new field of biotechnology called genetic code expansion (GCE). GCE is a technique used to produce proteins with the site-specific co-translational incorporation of additional non-canonical amino acids (ncAAs) beyond the 20 standard amino acids normally used in protein synthesis. Today, a wide variety of ncAAs with diverse functions can be installed by GCE, permitting many applications in a variety of expression hosts from bacteria to yeast to animal models. 

GCE enables the inclusion of unique functional groups into proteins and involves a range of applications including site-directed post-translational modifications (PTMs) with *N_ε_*-acetyl-lysine (acK) [124], *O*-phosphoserine (pSer) [125], *O*-phosphothreonine (pThr) [126,127], and *O*-phosphotyrosine (pTyr) [128]. Cross-linking ncAAs [129] allow studies of protein–protein interactions [130], while fluorescent ncAAs [131] enable imaging and localization studies [132] in live cells. Indeed, the mechanisms to produce selenoproteins covered earlier are important examples of GCE that have been fruitful systems for further engineering (see Section 5.4, Section 5.5, Section 5.6, Section 6 and Section 7).

The Pyl system has shown unparalleled flexibility and portability in terms of the chemical diversity of ncAAs and in its facile distribution to diverse expression host systems and model organisms [133]. Many of the PylRS/tRNA^Pyl^ pairs used for GCE were developed from the naturally occurring *M. barkeri* or *Methanosarcina mazei* PylRS/tRNA^Pyl^ systems [134]. Expression of the PylRS/tRNA^Pyl^ pair from *M. barkeri* allowed all instances of UAG to be decoded using Pyl analogues in *Escherichia coli* [135]. Multiple PylRS/tRNA^Pyl^ pairs from several species can decode UAG with Pyl analogues in *E. coli*, including PylRSs from *M. mazei* [136], *M. bakeri* [135], and *Methanomethylophilus alvus* [137]. These naturally occurring PylRS/tRNA^Pyl^ pairs are capable of inserting multiple different Pyl analogues in response to UAG codons in *E. coli* [138]. Several mutant variants were developed following the discovery of PylRS [138,139,140,141,142] to vastly increase the number of ncAAs that can be inserted to more than 200 [143], making the Pyl system the most versatile orthogonal translation system (OTS) known [139]. Expression of the *M. mazei* PylRS/tRNA^Pyl^ pair and its variants in mammalian cells [144,145] and mice [146] allows reassignment of UAG to ncAAs in clinically relevant model systems. 

### 5.3. Using the PylRS System to Produce Selenoproteins

Although the PylRS system is the most common OTS used for GCE, it is not commonly used for genetically encoding Sec. One report used a mutant form of the PylRS/tRNA^Pyl^ pair in *E. coli* to reassign UAG codons to insert a photocaged version of Sec (*N*-(*tert*-Butoxycarbonyl)-[(*R*,*S*)-1-{4′,5′-(methylenedioxy)-2′-nitrophenyl}ethyl]-l-selenocysteine) to produce an *E. coli* peptidyl-prolyl cis–trans isomerase B (PpiB) and a Zika virus NS2B-NS3 protease (ZiPro), both containing the photocaged Sec [147]. Following purification, the photocaged Sec was decaged by exposure to ultraviolet (UV) light to produce Sec-containing versions of these proteins [147]. Although this system functioned, the bulky photocaged Sec was speculated to interfere with protein folding [147], potentially limiting the use of this system for the general production of selenoproteins.

### 5.4. Using the E. coli Sec Insertion System to Produce Selenoproteins

**Figure 4:**
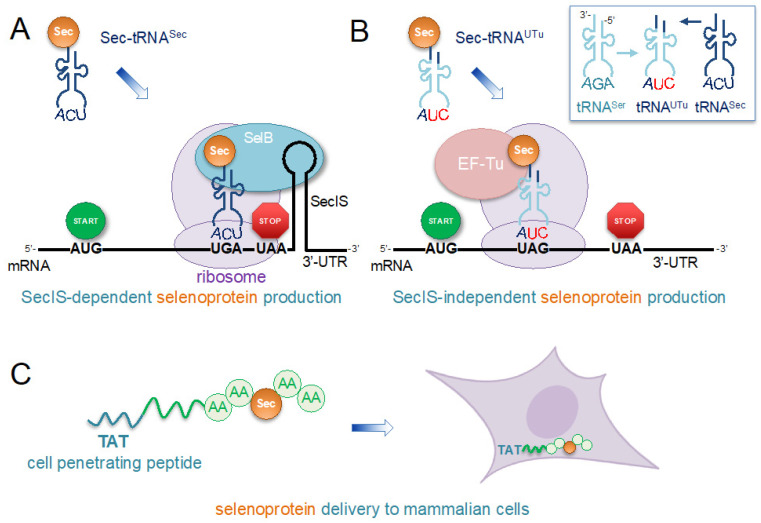
Engineering recombinant selenoprotein biosynthesis and delivery to cells. (**A**) The schematic shows an approach that we applied to efficiently produce human thioredoxin reductase 1 (TrxR1) in *E. coli* with stoichiometric incorporation of selenocysteine (Sec) in the active site [151]. Because the Sec codon is close to the untranslated region (UTR), a bacterial selenocysteine insertion sequence (SECIS) derived from the *E. coli* formate dehydrogenase gene was appended to the construct without perturbing the open reading frame. (**B**) For programmable and site-specific incorporation of Sec at any location in a recombinant protein that is independent of the SECIS element, a novel tRNA was designed to enable Sec incorporation using the normal elongation factor thermal unstable (EF-Tu) [156]. The tRNA contains the first 7 base pairs from the acceptor stem of tRNA^Sec^ (dark blue) transplanted in place of the first 6 base pairs in the body of tRNA^Ser^ (cyan). The resulting tRNA^UTu^ is aminoacylated with Ser and Ser-tRNA^UTu^ is converted to Sec-tRNA by selenocysteine synthase (SelA) (as in Figure 2). Because the tRNA includes a mutant anticodon (5′-CUA-3′), it reads the UAG stop codon to insert Sec. (**C**) Following the production of active human selenoproteins, we found that fusion with an N-terminal transactivator of transcription (TAT) cell-penetrating peptide tag enables efficient transduction of recombinant selenoprotein into the cytosol of human cells [200]. Thus, the approach enables the synthesis of engineered proteins in an efficient production host or synthetic cell and the ability to then investigate selenoproteins in the homologous context of otherwise naive human cells.

Most other techniques for translation with Sec rely on using variations of the naturally occurring systems, such as the *E. coli* Sec insertion system (Figure 2A). The dependence of the system on the presence of SECIS downstream of the recoding site, often in the ORF, to direct Sec insertion by binding SelB presents challenges to using this system for site-specific Sec insertion [148]. For some proteins with Sec occurring at the C-terminal, a bacterial SECIS can be inserted into the 3′-UTR to allow recombinant production without changes to the ORF [18,149] (Figure 4A). A mammalian selenoprotein was recombinantly produced by fusing an engineered bacterial SECIS element with the rat thioredoxin reductase (TrxR) gene without causing changes to the protein sequence [150]. Recombinant production of human TrxR was also achieved without changing the amino acid sequence with a similar method [149,151] (Figure 4A). 

For production in a bacterial host, other Sec-containing proteins with internal Sec residues require a SECIS present within the ORF. In one case, an artificial SECIS was engineered to minimize changes to the protein sequence encoded by the SECIS [18]. A Sec-containing glutathione S-transferase was produced recombinantly in *E. coli* by inserting a minimal bacterial SECIS from the *E. coli fdhF* gene downstream of the Sec codon, which resulted in six point mutations that had no effect on enzyme activity or substrate binding [152]. In another case, a Sec-containing human MsrB1 was produced by mutating the ORF downstream of the UGA Sec codon to generate a SECIS functional in *E. coli* [153]. This engineered SECIS mutated four residues in MsrB1, but these mutations also had little to no effect on enzyme activity [153]. 

### 5.5. SECIS-Independent Incorporation of Sec in Bacteria

Several engineering approaches overcame the need for SECIS in translation with Sec [154,155,156,157]. One technique used *E. coli* tRNA^Sec^ with a mutated anticodon and an engineered *E. coli* strain, C321.ΔA [154]. The C321.ΔA *E. coli* strain has all UAG stop codons mutated to UAA and a deletion of Release Factor 1 (RF1) [158], which terminates translation at UAG stop codons. By mutating the anticodon of tRNA^Sec^ to CUA, tRNA^Sec^ was capable of inserting Sec at UAG codons in the C321.ΔA *E. coli* strain without the need for SECIS [154]. However, this technique resulted in a heterogenous mix of protein products due to the mis-incorporation of glutamine and lysine at the UAG codon [154] and codon skipping at UAG [159], producing proteins lacking Sec [154,159]. 

A novel SECIS-independent approach to selenoprotein production was achieved by creating a chimera of tRNA^Ser^ and tRNA^Sec^ that interacts with EF-Tu instead of SelB [156] (Figure 4B). SelB typically binds Sec-tRNA^Sec^ to bring it to the ribosome by binding SECIS (Figure 2A), while EF-Tu is responsible for bringing all other elongator tRNAs to the ribosome (Figure 1B). A synthetic tRNA (tRNA^UTu^) was created by combining the backbone of tRNA^Ser^ with the acceptor helix of tRNA^Sec^ [156] (Figure 4B). The tRNA^UTu^ is a competent substrate for both SerRS and SelA [156,157]. This allowed tRNA^UTu^ to be charged with Ser by SerRS, followed by conversion of Ser-tRNA^UTu^ to Sec-tRNA^UTu^ by SelA, while being delivered to the ribosome by EF-Tu instead of SelB, eliminating the need for a downstream SECIS [156] (Figure 4B). The initial version of tRNA^UTu^ resulted in ~30% misincorporation of Ser, due to the incomplete conversion of Ser-tRNA^UTu^ to Sec-tRNA^UTu^ by SelA [156]. Further engineering of tRNA^UTu^ improved the interaction with SelA, producing tRNA^UTuX^, which was capable of stoichiometric incorporation of Sec without contamination with Ser [157]. 

An identity element in tRNA^Sec^ for *E. coli* SelA is a non-canonical 13-branch structure [160], consisting of the acceptor and T-stems. The *E. coli* tRNA^Sec^, tRNA^Utu^, and tRNA^UTuX^ all have a non-canonical 13-bp branch [161], which is efficiently recognized by SelB [162]. In contrast, EF-Tu mediated translation is optimal with tRNAs with the canonical 12-bp branch structure [163]. A new system for SECIS-independent selenoprotein production was developed using tRNAs with a 12-bp branch structure to improve compatibility with EF-Tu [161]. The system uses SelA from *Aeromonas almonicida* subspecies *pectinolytica* 34mel (*As*SelA) [161], which recognizes a 12-bp tRNA branch structure [30] along with a mutant Allo-tRNA [161]. 

Allo-tRNAs are a recently discovered group of tRNAs with unusual cloverleaf structures [164,165]. An allo-tRNA^Ser^ was identified in metagenomic databases that also included the identity elements for SelA [161]. Expression of *As*SelA and a UAG-decoding tRNA variant (allo-tRNA^Utu1^) in *E. coli* promoted the translation of 5 UAG codons as Sec in one polypeptide [161]. Allo-tRNA^Utu1^ was mutated to include a segment of the *A. salmonicida* tRNA^Sec^ D-stem (allo-tRNA^UTu1D^), leading to increased conversion from Ser-allo-tRNA^UTu1^ to Sec-allo-tRNA^UTu1^ by *As*SelA. *As*SelA was also engineered by mutagenesis and directed evolution to increase the efficiency with which *As*SelA converts Ser-tRNA^UTu1D^ to Sec-tRNA^UTu1D^, producing *As*SelA^Evol^. To further increase the supply of selenium, *Aeromonas almonicida* SelD (*As*SelD) and selenocysteine lyase SufS(C364A) were co-expressed, further increasing the efficiency of Sec insertion [161]. A single plasmid (pSecUAG-Evol2) encoding *As*SelA^Evol^, tRNA^UTu1D^, *As*SelD, and Sec lyase SufS(C364A) allowed the production of selenoprotein with a Sec incorporation stoichiometry of 90% [161]. A recent study demonstrated that site-specific Sec incorporation increased the O_2_ tolerance of a hydrogenase enzyme [166], and a review suggested many applications for this approach in generating Sec-containing hydrogenase enzymes for hydrogen production to meet the needs for clean and renewable sources of energy [167].

### 5.6. Genetically Encoded Sec in Yeast 

Most approaches for producing recombinant selenoproteins rely on *E. coli* as a production system. Some selenoproteins cannot be efficiently produced in bacteria [157]. To overcome this barrier, recent efforts engineered yeast to genetically encode Sec. Since yeast and related species lack the Sec-decoding trait, these approaches provide the first routes to produce selenoproteins in yeast. 

One approach used a mutant form of an orthogonal leucyl-tRNA synthetase (LeuRS)/tRNA^Leu^ pair that is capable of inserting a photocaged Sec (4,5-dimethyloxy-2-nitrobenzyl Sec, DMNB-Sec) in response to UAG codons to produce DMNB-Sec-containing proteins in yeast [168]. Following purification of DMNB-Sec-containing proteins, the DMNB caging group was removed by exposure to UV light, producing selenoprotein [168]. A second approach adapted the Sec incorporation machinery from bacteria for SECIS-independent insertion of Sec in response to UAG codons in *Saccharomyces cerevisiae* [169]. A *S. cerevisiae* tRNA^Ser^ was mutated to create a synthetic tRNA (*Sc*tRNA^Sec^) that was aminoacylated by the endogenous *S. cerevisiae* SerRS and then converted from Ser-*Sc*tRNA^Sec^ to Sec-*Sc*tRNA^Sec^ by *Aeromonas salmonicida* SelA (*As*SelA). The anticodon of *Sc*tRNA^Sec^ was also mutated to decode UAG codons [169]. An *A. salmonicida* SelD (*As*SelD) was also used to create selenophosphate from selenite, the selenium donor for the conversion of Ser-*Sc*tRNA^Sec^ to Sec-*Sc*tRNA^Sec^ by *As*SelA [169]. Additionally, selenite is converted to free Sec in *S. cerevisiae* via the Cys biosynthetic pathway [170], so a *Mus musculus* Sec lyase (*Mm*SCL) was added to convert free Sec back to selenite to ensure sufficient selenite availability for *As*SelD to produce selenophosphate [169]. A single plasmid expressing *Sc*tRNA^Sec^, *As*SelD, *As*SelA, and *Mm*SCL is sufficient to insert Sec at UAG codons, permitting selenoprotein production in *S. cerevisiae*, without the need for SECIS or SelB, which enabled the successful production of Sec-containing human selenoprotein, MsrB1, in yeast [169].

## 6. Challenges and Opportunities in Genetic Code Expansion with ncAAs and Sec

### 6.1. Codon Availability: A Limitation of Genetic Code Expansion

The availability of codons that can be reassigned to ncAAs remains a limiting factor for GCE, making the production of proteins simultaneously containing two or more ncAAs challenging or inefficient. GCE has mostly focused on using one of the three stop codons (UAG, UAA, or UGA) [171]. By mutating the tRNA^Pyl^ anticodon, the PylRS/tRNA^Pyl^ system can reassign any of the stop codons [172]. Indeed, the production of a single protein containing two ncAAs incorporated via GCE simultaneously in *E. coli* was achieved using the Pyl system to reassign UAA stop codons and using the *M. jannaschii* tyrosyl-tRNA synthetase (TyrRS) system to reassign UAG stop codons [173]. The approach used three stop codons in the ORF with two of the three stop codons being used to insert ncAAs, and the third (UGA) to direct translational termination. 

So far, up to three different ncAAs have been simultaneously inserted site-specifically into a single polypeptide chain in *E. coli* by either reassigning all three stop codons [174] or by reassigning two stop codons and using UAU as a start codon (instead of AUG) to insert a ncAA at the N-terminus of a protein using an engineered initiator tRNA [175]. To reassign all three stop codons, a PylRS/tRNA^Pyl^_UUA_ pair decoded UAA to Boc-lysine (BocK), *E. coli* tryptophanyl-tRNA synthetase (TrpRS)/tRNA^Trp^_UCA_ decoded UGA to 5-hydroxytryptophan, and *M. jannaschii* TyrRS/tRNA^Tyr^_CUA_ decoded UAG to p-azido-phenylalanine in the engineered *E. coli* strain ATMW1 [174]. The ATMW1 strain has the endogenous *E. coli* TrpRS/tRNA^Trp^ substituted with its yeast counterpart to allow the insertion of ncAA with the *E. coli* TrpRS/tRNA^Trp^ pair [176]. Translational termination was achieved by multiple consecutive stop codons even in the presence of an orthogonal pair capable of decoding the stop codon [177]. Three consecutive UAA stop codons were used to direct translational termination, and a self-cleaving tag was used to remove partial ncAA insertion by the PylRS/tRNA^Pyl^_UUA_ at the C-terminal translational termination site [174]. 

Drawbacks to this system include the lack of a dedicated stop codon resulting in the need for a complicated self-cleaving tag to ensure C-terminus homogeneity [174] and the need for an engineered *E. coli* strain [174] which may be difficult to duplicate in other host systems. Another challenge is the low yield of ncAA-containing proteins, e.g., proteins containing three ncAAs were produced with only 2% of the yield of wildtype protein [174], which may result from competition of the orthogonal tRNAs with endogenous release factors, or toxicity from elongated endogenous proteins produced due to endogenous stop codon readthrough, as observed in a GCE system for phosphoserine [178]. 

### 6.2. Sense Codon Reassignment 

Due to the degeneracy of the genetic code, it is widely believed that many sense codons, perhaps 20 or more, should be available for recoding or reassignment to ncAAs [171]. Indeed, the Pyl system was used in an attempt to reassign the CGG arginine (Arg) codon in *Mycoplasma capricolum* [171]. The CGG codon in *M. capricolum* has been called an unused or “open” sense codon [179] because the genome only contains six CGG arginine (Arg) codons and lacks a tRNA dedicated to decoding CGG [171]. Unfortunately, the expression of PylRS and tRNA^Pyl^ with a CCG anticodon (tRNA^Pyl^_CCG_) in *M. capricolum* resulted in the loading of tRNA^Pyl^_CCG_ with Arg by endogenous ArgRS and decoding of CGG as Arg [171]. The tRNA^Pyl^ variants with CCG anticodons are aminoacylated in *E. coli* by ArgRS while ArgRS was not active with tRNA^Pyl^_CUA_ or tRNA^Pyl^_GAG_ [171], suggesting that cross-reactivity with endogenous translation machinery may prove to be an additional barrier when attempting to decode sense codons. A comprehensive study of sense codon reassignment in *E. coli* found that orthogonal *M. jannaschii* TyrRS [180] and *M. bakeri* PylRS [181] pairs could effectively outcompete many sense codons, providing up to 65% missense suppression of the Arg AGG codon with an ncAA.

The *E. coli* Sec insertion system provides the molecular machinery to bypass barriers to installing ncAAs at sense codons. An interesting aspect of the *E. coli* Sec insertion system is the ability to change the meaning of sense codons. By simply changing the tRNA^Sec^ anticodon, Sec can be inserted at 58 of the 64 possible codons with some efficiency, and can completely convert all three stop codons and 15 different sense codons to encode Sec [149]. Fascinatingly, in nature, some species use Cys codons and other sense codons for recoding to Sec [30]. By combining the Sec system with other OTSs, like the PylRS system, many more ncAAs could be inserted into a single polypeptide chain.

### 6.3. Combining the E. coli Sec Insertion System with the PylRS/tRNA^Pyl^ System

The Sec insertion system was combined with the PylRS/tRNA^Pyl^ system to produce a protein with 22 amino acids, including two ncAAs, Sec and acetyl-lysine (acK), in *E. coli*. A Sec-containing human TrxR1 site-specifically acetylated at experimentally identified acetylation sites was produced recombinantly in *E. coli* [151]. A bacterial SECIS was included in the 3′-UTR of TrxR1 to direct the *E. coli* Sec insertion machinery to recode a UGA codon to Sec (Figure 4A), while simultaneously reassigning UAG codons to acK using a mutant PylRS/tRNA^Pyl^ pair [151]. Biochemical characterization of the acetylated, Sec-containing TrxR1 variants demonstrated that acetylation increased TrxR1 activity by destabilizing low-activity tetramers [151]. The work demonstrated that the *E. coli* Sec insertion system is compatible with the PylRS/tRNA^Pyl^ pair and could be used to study the post-translational modifications of selenoproteins. 

Recently, a related approach was established to generate selenoproteins using the nonsense suppressor Allo-tRNA^UTu1D^ system to install Sec at any desired position in combination with the acK-specific PylRS/tRNA^Pyl^ pair to produce site-specifically acetylated selenoproteins [182]. Fascinatingly, the UGA codon was used to encode AcK and UAG was used for Sec in initial experiments; however, swapping the codon assignments and using UAG for acK and UGA for Sec produced a more selective dual incorporation system. The method was applied to generate acetylated variants of the human GPx1. In the future, combining the *E. coli* Sec insertion system and the PylRS/tRNA^Pyl^ pair with other OTSs, such as the *M. jannaschii* TyrRS/tRNA^Tyr^ pair, could allow the incorporation of many more ncAAs into proteins. 

## 7. Overexpression and Delivery of Selenoproteins in Mammalian Cells 

### 7.1. Challenges in Selenoprotein Overexpression in Mammalian Cells

Overexpression of selenoproteins in mammalian cells from plasmids can be difficult due to the complicated and inefficient Sec insertion machinery. Overexpression of TrxR1 can also cause increased cell death in mammalian cells. TrxR1 overexpression from stable plasmid transfection caused more than double the amount of cell death in Michigan cancer foundation 7 (MCF-7) cells relative to an empty vector control [183]. TrxR1 lacking the Sec residue, due to a translational termination at the Sec-encoding UGA, is toxic to A549 cells and resulted in cell death when delivered by lipid-based transfection methods [184]. Selenium compromised TRxR-derived apoptotic proteins (SecTRAPS) [185], which are TrxR proteins either lacking the Sec residue or that have had the Sec residue derivatized with chemical compounds, may explain these results. SecTRAPs can also be created in mammalian cells by inhibition of endogenous TrxR1 with compounds that target the Sec residue, which also leads to apoptosis [186]. Transient transfection of plasmids containing DIO1, DIO2, or DIO3, with their corresponding SECIS sequences in the 3′-UTR, allowed successful production of full-length DIO1 and DIO3, but not DIO2 in HEK 293T cells [187]. Interestingly, relatively low levels of Sec-containing DIO1 and DIO3 were produced compared to HEK 293T cells transfected with plasmids containing Sec-to-Cys mutants, and high levels of DIO1 and DIO3 truncated at the UGA codon were observed [187]. Additionally, no experiments to validate Sec incorporation into the full-length DIO proteins were conducted. Thus, transient transfection approaches for DIO1 were not toxic, but they were inefficient or unable to produce the selenoproteins.

### 7.2. Opportunities to Investigate Selenoproteins in Mammalian Cells

Overexpression of other selenoproteins in mammalian cells has also proven challenging. Overexpression of the selenoprotein GPx1 from a plasmid in endothelial cells required co-transfection of SelD and tRNA^Sec^ [188], while overexpression of GPx3 in mammalian cells required co-transfection of SelD, tRNA^Sec^, and SBP2 [189]. A plasmid developed for mammalian expression of selenoproteins (pCI-HHT-Toxo-SECIS vector) contains a highly efficient SECIS from *Toxoplasma gondii* in the 3′-UTR of the selenoprotein gene of interest and also co-expresses SBP2 [190]. The plasmid has been used for the expression of selenoprotein S [113], selenoprotein K [114], selenoprotein O [110], and GPx4 [191] in mammalian cells. Each of these techniques required overexpression of SBP2 or other proteins involved in Sec insertion, which may alter the cellular phenotype, potentially complicating any observations related to the activity of the selenoprotein itself. 

### 7.3. Cell-Penetrating Peptide for Delivery of the Selenoproteins TrxR1 to Live Cells

Cell-penetrating peptides (CPPs) are small peptides that cross cell membranes and allow the delivery of attached cargo, such as mRNAs [192], proteins [193], and small molecules [194], to mammalian cells [195]. One such CPP is derived from the human immunodeficiency virus protein, transactivator of transcription (TAT) [196,197,198]. The TAT protein has a small basic domain which transverses cell membranes to achieve cellular uptake of the covalently attached cargo molecules [199]. The TAT-tag has been used to deliver recombinant proteins into various types of mammalian and plant cells [199]. 

Recently, Sec-containing human TrxR1 that was produced recombinantly in *E. coli* with GCE was delivered to the cytoplasm of mammalian cells using an N-terminal TAT-tag [200]. Sec-insertion into TrxR1 was achieved in *E. coli* using a bacterial SECIS in the 3′-UTR to direct Sec insertion at UGA, with a TAT-tag fused to the N-terminus [200] (Figure 4A). Following purification of TAT-TrxR1 and incubation with HEK 293T cells, a live-cell and TrxR-specific activity reporter was used to confirm the successful delivery of active and Sec-containing TrxR1 to the cytoplasm of human cells without the need for any lipid-based transfection reagents (Figure 4C) [200]. This new approach to delivering selenoproteins to mammalian cells with a CPP could be applied to other natural or synthetic selenoproteins. The method avoids the need to overexpress components of the Sec insertion system in the cell where the selenoprotein of interest is under investigation. Selenoproteins fused to a CPP can be produced in the *E. coli* or yeast GCE systems for Sec noted above. Following purification, these CPP-linked selenoproteins can be characterized biochemically and delivered directly to cells (Figure 4C) to investigate the biological function of selenoproteins in the homologous context of live mammalian cells.

## References

[1] Crick F.H. (1958). On protein synthesis. Symp. Soc. Exp. Biol..

[2] Brenner S., Jacob F., Meselson M. (1961). An unstable intermediate carrying information from genes to ribosomes for protein synthesis. Nature.

[3] Ramakrishnan V. (2002). Ribosome structure and the mechanism of translation. Cell.

[4] Nirenberg M., Leder P., Bernfield M., Brimacombe R., Trupin J., Rottman F., O’Neal C. (1965). RNA codewords and protein synthesis, VII. On the general nature of the RNA code. Proc Natl. Acad. Sci. USA.

[5] Söll D., Ohtsuka E., Jones D.S., Lohrmann R., Hayatsu H., Nishimura S., Khorana H.G. (1965). Studies on polynucleotides, XLIX. Stimulation of the binding of aminoacyl-sRNA’s to ribosomes by ribotrinucleotides and a survey of codon assignments for 20 amino acids. Proc. Natl. Acad. Sci. USA.

[6] Crick F.H., Barnett L., Brenner S., Watts-Tobin R.J. (1961). General nature of the genetic code for proteins. Nature.

[7] Ibba M., Söll D. (1999). Quality control mechanisms during translation. Science.

[8] Ibba M., Curnow A.W., Söll D. (1997). Aminoacyl-tRNA synthesis: Divergent routes to a common goal. Trends Biochem. Sci..

[9] Yadavalli S.S., Ibba M. (2012). Quality control in aminoacyl-tRNA synthesis its role in translational fidelity. Adv. Protein Chem. Struct. Biol..

[10] Giegé R., Sissler M., Florentz C. (1998). Universal rules and idiosyncratic features in tRNA identity. Nucleic Acids Res..

[11] Giegé R., Eriani G. (2023). The tRNA identity landscape for aminoacylation and beyond. Nucleic Acids Res..

[12] Mukai T., Lajoie M.J., Englert M., Söll D. (2017). Rewriting the Genetic Code. Annu. Rev. Microbiol..

[13] Böck A., Forchhammer K., Heider J., Baron C. (1991). Selenoprotein synthesis: An expansion of the genetic code. Trends Biochem. Sci..

[14] Commans S., Böck A. (1999). Selenocysteine inserting tRNAs: An overview. FEMS Microbiol. Rev..

[15] Rother M., Quitzke V. (2018). Selenoprotein synthesis and regulation in Archaea. Biochim. Biophys. Acta Gen. Subj..

[16] Peng J.J., Yue S.Y., Fang Y.H., Liu X.L., Wang C.H. (2021). Mechanisms Affecting the Biosynthesis and Incorporation Rate of Selenocysteine. Molecules.

[17] Lobanov A.V., Hatfield D.L., Gladyshev V.N. (2009). Eukaryotic selenoproteins and selenoproteomes. Biochim. Biophys. Acta.

[18] Chung C.Z., Krahn N. (2022). The selenocysteine toolbox: A guide to studying the 21st amino acid. Arch. Biochem. Biophys..

[19] Schmidt R.L., Simonović M. (2012). Synthesis and decoding of selenocysteine and human health. Croat. Med. J..

[20] Xu X.M., Carlson B.A., Irons R., Mix H., Zhong N., Gladyshev V.N., Hatfield D.L. (2007). Selenophosphate synthetase 2 is essential for selenoprotein biosynthesis. Biochem. J..

[21] Forchhammer K., Leinfelder W., Boesmiller K., Veprek B., Böck A. (1991). Selenocysteine synthase from *Escherichia coli.* Nucleotide sequence of the gene (selA) and purification of the protein. J. Biol. Chem..

[22] Carlson B.A., Xu X.M., Kryukov G.V., Rao M., Berry M.J., Gladyshev V.N., Hatfield D.L. (2004). Identification and characterization of phosphoseryl-tRNA[Ser]Sec kinase. Proc. Natl. Acad. Sci. USA.

[23] Sherrer R.L., O’Donoghue P., Söll D. (2008). Characterization and evolutionary history of an archaeal kinase involved in selenocysteinyl-tRNA formation. Nucleic Acids Res..

[24] Lee B.J., Worland P.J., Davis J.N., Stadtman T.C., Hatfield D.L. (1989). Identification of a selenocysteyl-tRNA(Ser) in mammalian cells that recognizes the nonsense codon. UGA. J. Biol. Chem..

[25] Yuan J., Palioura S., Salazar J.C., Su D., O’Donoghue P., Hohn M.J., Cardoso A.M., Whitman W.B., Söll D. (2006). RNA-dependent conversion of phosphoserine forms selenocysteine in eukaryotes and archaea. Proc. Natl. Acad. Sci. USA.

[26] Xu X.M., Carlson B.A., Mix H., Zhang Y., Saira K., Glass R.S., Berry M.J., Gladyshev V.N., Hatfield D.L. (2007). Biosynthesis of selenocysteine on its tRNA in eukaryotes. PLoS Biol..

[27] Araiso Y., Palioura S., Ishitani R., Sherrer R.L., O’Donoghue P., Yuan J., Oshikane H., Domae N., Defranco J., Söll D. (2008). Structural insights into RNA-dependent eukaryal and archaeal selenocysteine formation. Nucleic Acids Res..

[28] Zhang Y., Gladyshev V.N. (2005). An algorithm for identification of bacterial selenocysteine insertion sequence elements and selenoprotein genes. Bioinformatics.

[29] Krol A. (2002). Evolutionarily different RNA motifs and RNA-protein complexes to achieve selenoprotein synthesis. Biochimie.

[30] Mukai T., Englert M., Tripp H.J., Miller C., Ivanova N.N., Rubin E.M., Kyrpides N.C., Söll D. (2016). Facile Recoding of Selenocysteine in Nature. Angew. Chem. Int. Ed. Engl..

[31] Ringquist S., Schneider D., Gibson T., Baron C., Böck A., Gold L. (1994). Recognition of the mRNA selenocysteine insertion sequence by the specialized translational elongation factor SELB. Genes Dev..

[32] Zavacki A.M., Mansell J.B., Chung M., Klimovitsky B., Harney J.W., Berry M.J. (2003). Coupled tRNA(Sec)-dependent assembly of the selenocysteine decoding apparatus. Mol. Cell.

[33] Rother M., Wilting R., Commans S., Böck A. (2000). Identification and characterisation of the selenocysteine-specific translation factor SelB from the archaeon *Methanococcus jannaschii*. J. Mol. Biol..

[34] Fletcher J.E., Copeland P.R., Driscoll D.M., Krol A. (2001). The selenocysteine incorporation machinery: Interactions between the SECIS RNA and the SECIS-binding protein SBP2. RNA.

[35] Meng K., Chung C.Z., Söll D., Krahn N. (2022). Unconventional genetic code systems in archaea. Front. Microbiol..

[36] Peng T., Lin J., Xu Y.Z., Zhang Y. (2016). Comparative genomics reveals new evolutionary and ecological patterns of selenium utilization in bacteria. ISME J..

[37] Hoffmann P.R., Berry M.J. (2005). Selenoprotein synthesis: A unique translational mechanism used by a diverse family of proteins. Thyroid.

[38] Kryukov G.V., Gladyshev V.N. (2004). The prokaryotic selenoproteome. EMBO Rep..

[39] Mariotti M., Ridge P.G., Zhang Y., Lobanov A.V., Pringle T.H., Guigo R., Hatfield D.L., Gladyshev V.N. (2012). Composition and evolution of the vertebrate and mammalian selenoproteomes. PLoS ONE.

[40] Chapple C.E., Guigo R. (2008). Relaxation of selective constraints causes independent selenoprotein extinction in insect genomes. PLoS ONE.

[41] Mariotti M., Salinas G., Gabaldon T., Gladyshev V.N. (2019). Utilization of selenocysteine in early-branching fungal phyla. Nat. Microbiol..

[42] Novoselov S.V., Rao M., Onoshko N.V., Zhi H., Kryukov G.V., Xiang Y., Weeks D.P., Hatfield D.L., Gladyshev V.N. (2002). Selenoproteins and selenocysteine insertion system in the model plant cell system, Chlamydomonas reinhardtii. EMBO J..

[43] Fu L.H., Wang X.F., Eyal Y., She Y.M., Donald L.J., Standing K.G., Ben-Hayyim G. (2002). A selenoprotein in the plant kingdom. Mass spectrometry confirms that an opal codon (UGA) encodes selenocysteine in Chlamydomonas reinhardtii gluththione peroxidase. J. Biol. Chem..

[44] Lobanov A.V., Fomenko D.E., Zhang Y., Sengupta A., Hatfield D.L., Gladyshev V.N. (2007). Evolutionary dynamics of eukaryotic selenoproteomes: Large selenoproteomes may associate with aquatic life and small with terrestrial life. Genome Biol..

[45] Kryukov G.V., Castellano S., Novoselov S.V., Lobanov A.V., Zehtab O., Guigo R., Gladyshev V.N. (2003). Characterization of mammalian selenoproteomes. Science.

[46] Papp L.V., Lu J., Holmgren A., Khanna K.K. (2007). From selenium to selenoproteins: Synthesis, identity, and their role in human health. Antioxid. Redox Signal..

[47] Lu J., Holmgren A. (2009). Selenoproteins. J. Biol. Chem..

[48] Lu J., Holmgren A. (2014). The thioredoxin antioxidant system. Free Radic. Biol. Med..

[49] Cnubben N.H., Rietjens I.M., Wortelboer H., van Zanden J., van Bladeren P.J. (2001). The interplay of glutathione-related processes in antioxidant defense. Environ. Toxicol. Pharmacol..

[50] Brigelius-Flohe R., Flohe L. (2020). Regulatory Phenomena in the Glutathione Peroxidase Superfamily. Antioxid. Redox Signal..

[51] Pei J., Pan X., Wei G., Hua Y. (2023). Research progress of glutathione peroxidase family (GPX) in redoxidation. Front. Pharmacol..

[52] Hatfield D.L., Tsuji P.A., Carlson B.A., Gladyshev V.N. (2014). Selenium and selenocysteine: Roles in cancer, health, and development. Trends Biochem. Sci..

[53] Labunskyy V.M., Hatfield D.L., Gladyshev V.N. (2014). Selenoproteins: Molecular pathways and physiological roles. Physiol. Rev..

[54] Johansson L., Gafvelin G., Arnér E.S. (2005). Selenocysteine in proteins-properties and biotechnological use. Biochim. Et Biophys. Acta.

[55] Arnér E.S. (2010). Selenoproteins-What unique properties can arise with selenocysteine in place of cysteine?. Exp. Cell Res..

[56] Zhong L., Holmgren A. (2000). Essential role of selenium in the catalytic activities of mammalian thioredoxin reductase revealed by characterization of recombinant enzymes with selenocysteine mutations. J. Biol. Chem..

[57] Lee S.R., Bar-Noy S., Kwon J., Levine R.L., Stadtman T.C., Rhee S.G. (2000). Mammalian thioredoxin reductase: Oxidation of the C-terminal cysteine/selenocysteine active site forms a thioselenide, and replacement of selenium with sulfur markedly reduces catalytic activity. Proc. Natl. Acad. Sci. USA.

[58] Scalcon V., Bindoli A., Rigobello M.P. (2018). Significance of the mitochondrial thioredoxin reductase in cancer cells: An update on role, targets and inhibitors. Free Radic. Biol. Med..

[59] Zhang J., Zhang B., Li X., Han X., Liu R., Fang J. (2019). Small molecule inhibitors of mammalian thioredoxin reductase as potential anticancer agents: An update. Med. Res. Rev..

[60] Yang Y., Sun S., Xu W., Zhang Y., Yang R., Ma K., Zhang J., Xu J. (2022). Piperlongumine Inhibits Thioredoxin Reductase 1 by Targeting Selenocysteine Residues and Sensitizes Cancer Cells to Erastin. Antioxidants.

[61] Urig S., Becker K. (2006). On the potential of thioredoxin reductase inhibitors for cancer therapy. Semin. Cancer Biol..

[62] Gromer S., Urig S., Becker K. (2004). The thioredoxin system--from science to clinic. Med. Res. Rev..

[63] Onodera T., Momose I., Kawada M. (2019). Potential Anticancer Activity of Auranofin. Chem. Pharm. Bull..

[64] Hirt R.P., Muller S., Embley T.M., Coombs G.H. (2002). The diversity and evolution of thioredoxin reductase: New perspectives. Trends Parasitol..

[65] Arnér E.S. (2009). Focus on mammalian thioredoxin reductases--important selenoproteins with versatile functions. Biochim. Biophys. Acta.

[66] Tamura T., Stadtman T.C. (1996). A new selenoprotein from human lung adenocarcinoma cells: Purification, properties, and thioredoxin reductase activity. Proc. Natl. Acad. Sci. USA.

[67] Luthman M., Holmgren A. (1982). Rat liver thioredoxin and thioredoxin reductase: Purification and characterization. Biochemistry.

[68] Arnér E.S., Zhong L., Holmgren A. (1999). Preparation and assay of mammalian thioredoxin and thioredoxin reductase. Methods Enzymol..

[69] Chae H.Z., Kim H.J., Kang S.W., Rhee S.G. (1999). Characterization of three isoforms of mammalian peroxiredoxin that reduce peroxides in the presence of thioredoxin. Diabetes Res. Clin. Pract..

[70] Wood Z.A., Schroder E., Robin Harris J., Poole L.B. (2003). Structure, mechanism and regulation of peroxiredoxins. Trends Biochem. Sci..

[71] Kang S.W., Chae H.Z., Seo M.S., Kim K., Baines I.C., Rhee S.G. (1998). Mammalian peroxiredoxin isoforms can reduce hydrogen peroxide generated in response to growth factors and tumor necrosis factor-alpha. J. Biol. Chem..

[72] Peshenko I.V., Shichi H. (2001). Oxidation of active center cysteine of bovine 1-Cys peroxiredoxin to the cysteine sulfenic acid form by peroxide and peroxynitrite. Free Radic. Biol. Med..

[73] Dubuisson M., Vander Stricht D., Clippe A., Etienne F., Nauser T., Kissner R., Koppenol W.H., Rees J.F., Knoops B. (2004). Human peroxiredoxin 5 is a peroxynitrite reductase. FEBS Lett..

[74] Chae H.Z., Robison K., Poole L.B., Church G., Storz G., Rhee S.G. (1994). Cloning and sequencing of thiol-specific antioxidant from mammalian brain: Alkyl hydroperoxide reductase and thiol-specific antioxidant define a large family of antioxidant enzymes. Proc. Natl. Acad. Sci. USA.

[75] Bjornstedt M., Hamberg M., Kumar S., Xue J., Holmgren A. (1995). Human thioredoxin reductase directly reduces lipid hydroperoxides by NADPH and selenocystine strongly stimulates the reaction via catalytically generated selenols. J. Biol. Chem..

[76] Das K.C., Das C.K. (2000). Thioredoxin, a singlet oxygen quencher and hydroxyl radical scavenger: Redox independent functions. Biochem. Biophys. Res. Commun..

[77] Holmgren A., Lu J. (2010). Thioredoxin and thioredoxin reductase: Current research with special reference to human disease. Biochem. Biophys. Res. Commun..

[78] Lu J., Holmgren A. (2012). Thioredoxin system in cell death progression. Antioxid. Redox Signal..

[79] Powis G., Mustacich D., Coon A. (2000). The role of the redox protein thioredoxin in cell growth and cancer. Free Radic. Biol. Med..

[80] Zhang Y., Roh Y.J., Han S.J., Park I., Lee H.M., Ok Y.S., Lee B.C., Lee S.R. (2020). Role of Selenoproteins in Redox Regulation of Signaling and the Antioxidant System: A Review. Antioxidants.

[81] Mahmood D.F., Abderrazak A., El Hadri K., Simmet T., Rouis M. (2013). The thioredoxin system as a therapeutic target in human health and disease. Antioxid. Redox Signal..

[82] Liu Y., Xue N., Zhang B., Lv H., Li S. (2022). Role of Thioredoxin-1 and its inducers in human health and diseases. Eur. J. Pharmacol..

[83] Lillig C.H., Holmgren A. (2007). Thioredoxin and related molecules–from biology to health and disease. Antioxid. Redox Signal..

[84] Lovell M.A., Xie C., Gabbita S.P., Markesbery W.R. (2000). Decreased thioredoxin and increased thioredoxin reductase levels in Alzheimer’s disease brain. Free Radic. Biol. Med..

[85] Maurice M.M., Nakamura H., Gringhuis S., Okamoto T., Yoshida S., Kullmann F., Lechner S., van der Voort E.A., Leow A., Versendaal J. (1999). Expression of the thioredoxin-thioredoxin reductase system in the inflamed joints of patients with rheumatoid arthritis. Arthritis Rheumatol..

[86] Yamada Y., Nakamura H., Adachi T., Sannohe S., Oyamada H., Kayaba H., Yodoi J., Chihara J. (2003). Elevated serum levels of thioredoxin in patients with acute exacerbation of asthma. Immunol. Lett..

[87] Whayne T.F., Parinandi N., Maulik N. (2015). Thioredoxins in cardiovascular disease. Can. J. Physiol. Pharmacol..

[88] Jia J.J., Geng W.S., Wang Z.Q., Chen L., Zeng X.S. (2019). The role of thioredoxin system in cancer: Strategy for cancer therapy. Cancer Chemother. Pharmacol..

[89] Soini Y., Kahlos K., Napankangas U., Kaarteenaho-Wiik R., Saily M., Koistinen P., Paaakko P., Holmgren A., Kinnula V.L. (2001). Widespread expression of thioredoxin and thioredoxin reductase in non-small cell lung carcinoma. Clin. Cancer Res..

[90] Lichtenfels R., Kellner R., Atkins D., Bukur J., Ackermann A., Beck J., Brenner W., Melchior S., Seliger B. (2003). Identification of metabolic enzymes in renal cell carcinoma utilizing PROTEOMEX analyses. Biochim. Biophys. Acta.

[91] Lincoln D.T., Al-Yatama F., Mohammed F.M., Al-Banaw A.G., Al-Bader M., Burge M., Sinowatz F., Singal P.K. (2010). Thioredoxin and thioredoxin reductase expression in thyroid cancer depends on tumour aggressiveness. Anticancer Res..

[92] Hedley D., Pintilie M., Woo J., Nicklee T., Morrison A., Birle D., Fyles A., Milosevic M., Hill R. (2004). Up-regulation of the redox mediators thioredoxin and apurinic/apyrimidinic excision (APE)/Ref-1 in hypoxic microregions of invasive cervical carcinomas, mapped using multispectral, wide-field fluorescence image analysis. Am. J. Pathol..

[93] Raffel J., Bhattacharyya A.K., Gallegos A., Cui H., Einspahr J.G., Alberts D.S., Powis G. (2003). Increased expression of thioredoxin-1 in human colorectal cancer is associated with decreased patient survival. J. Lab. Clin. Med..

[94] Roh J.L., Jang H., Kim E.H., Shin D. (2017). Targeting of the Glutathione, Thioredoxin, and Nrf2 Antioxidant Systems in Head and Neck Cancer. Antioxid. Redox Signal..

[95] Selenius M., Rundlof A.K., Olm E., Fernandes A.P., Bjornstedt M. (2010). Selenium and the selenoprotein thioredoxin reductase in the prevention, treatment and diagnostics of cancer. Antioxid. Redox Signal..

[96] Dong C., Zhang L., Sun R., Liu J., Yin H., Li X., Zheng X., Zeng H. (2016). Role of thioredoxin reductase 1 in dysplastic transformation of human breast epithelial cells triggered by chronic oxidative stress. Sci. Rep..

[97] Wang L., Yang Z., Fu J., Yin H., Xiong K., Tan Q., Jin H., Li J., Wang T., Tang W. (2012). Ethaselen: A potent mammalian thioredoxin reductase 1 inhibitor and novel organoselenium anticancer agent. Free Radic. Biol. Med..

[98] Brent G.A. (2012). Mechanisms of thyroid hormone action. J. Clin. Investig..

[99] Gromer S., Eubel J.K., Lee B.L., Jacob J. (2005). Human selenoproteins at a glance. Cell. Mol. Life Sci..

[100] St Germain D.L., Galton V.A., Hernandez A. (2009). Minireview: Defining the roles of the iodothyronine deiodinases: Current concepts and challenges. Endocrinology.

[101] Galton V.A. (2005). The roles of the iodothyronine deiodinases in mammalian development. Thyroid.

[102] Darras V.M., Van Herck S.L. (2012). Iodothyronine deiodinase structure and function: From ascidians to humans. J. Endocrinol..

[103] Hernandez A., Martinez M.E., Ng L., Forrest D. (2021). Thyroid Hormone Deiodinases: Dynamic Switches in Developmental Transitions. Endocrinology.

[104] Ng L., Hernandez A., He W., Ren T., Srinivas M., Ma M., Galton V.A., St Germain D.L., Forrest D. (2009). A protective role for type 3 deiodinase, a thyroid hormone-inactivating enzyme, in cochlear development and auditory function. Endocrinology.

[105] Ng L., Goodyear R.J., Woods C.A., Schneider M.J., Diamond E., Richardson G.P., Kelley M.W., Germain D.L., Galton V.A., Forrest D. (2004). Hearing loss and retarded cochlear development in mice lacking type 2 iodothyronine deiodinase. Proc. Natl. Acad. Sci. USA.

[106] Low S.C., Harney J.W., Berry M.J. (1995). Cloning and functional characterization of human selenophosphate synthetase, an essential component of selenoprotein synthesis. J. Biol. Chem..

[107] Guimaraes M.J., Peterson D., Vicari A., Cocks B.G., Copeland N.G., Gilbert D.J., Jenkins N.A., Ferrick D.A., Kastelein R.A., Bazan J.F. (1996). Identification of a novel selD homolog from eukaryotes, bacteria, and archaea: Is there an autoregulatory mechanism in selenocysteine metabolism?. Proc. Natl. Acad. Sci. USA.

[108] Saito Y. (2021). Selenium Transport Mechanism via Selenoprotein P-Its Physiological Role and Related Diseases. Front. Nutr..

[109] Tsuji P.A., Santesmasses D., Lee B.J., Gladyshev V.N., Hatfield D.L. (2021). Historical Roles of Selenium and Selenoproteins in Health and Development: The Good, the Bad and the Ugly. Int. J. Mol. Sci..

[110] Han S.J., Lee B.C., Yim S.H., Gladyshev V.N., Lee S.R. (2014). Characterization of mammalian selenoprotein o: A redox-active mitochondrial protein. PLoS ONE.

[111] Dikiy A., Novoselov S.V., Fomenko D.E., Sengupta A., Carlson B.A., Cerny R.L., Ginalski K., Grishin N.V., Hatfield D.L., Gladyshev V.N. (2007). SelT, SelW, SelH, and Rdx12: Genomics and molecular insights into the functions of selenoproteins of a novel thioredoxin-like family. Biochemistry.

[112] Horibata Y., Hirabayashi Y. (2007). Identification and characterization of human ethanolaminephosphotransferase1. J. Lipid Res..

[113] Turanov A.A., Shchedrina V.A., Everley R.A., Lobanov A.V., Yim S.H., Marino S.M., Gygi S.P., Hatfield D.L., Gladyshev V.N. (2014). Selenoprotein S is involved in maintenance and transport of multiprotein complexes. Biochem. J..

[114] Shchedrina V.A., Everley R.A., Zhang Y., Gygi S.P., Hatfield D.L., Gladyshev V.N. (2011). Selenoprotein K binds multiprotein complexes and is involved in the regulation of endoplasmic reticulum homeostasis. J. Biol. Chem..

[115] Ren B., Liu M., Ni J., Tian J. (2018). Role of Selenoprotein F in Protein Folding and Secretion: Potential Involvement in Human Disease. Nutrients.

[116] Reeves M.A., Bellinger F.P., Berry M.J. (2010). The neuroprotective functions of selenoprotein M and its role in cytosolic calcium regulation. Antioxid. Redox Signal..

[117] Hao B., Gong W., Ferguson T.K., James C.M., Krzycki J.A., Chan M.K. (2002). A new UAG-encoded residue in the structure of a methanogen methyltransferase. Science.

[118] Soares J.A., Zhang L., Pitsch R.L., Kleinholz N.M., Jones R.B., Wolff J.J., Amster J., Green-Church K.B., Krzycki J.A. (2005). The residue mass of L-pyrrolysine in three distinct methylamine methyltransferases. J. Biol. Chem..

[119] Mahapatra A., Patel A., Soares J.A., Larue R.C., Zhang J.K., Metcalf W.W., Krzycki J.A. (2006). Characterization of a Methanosarcina acetivorans mutant unable to translate UAG as pyrrolysine. Mol. Microbiol..

[120] O’Donoghue P., Prat L., Kucklick M., Schafer J.G., Riedel K., Rinehart J., Söll D., Heinemann I.U. (2014). Reducing the genetic code induces massive rearrangement of the proteome. Proc. Natl. Acad. Sci. USA.

[121] Krzycki J.A. (2005). The direct genetic encoding of pyrrolysine. Curr Opin Microbiol.

[122] Srinivasan G., James C.M., Krzycki J.A. (2002). Pyrrolysine encoded by UAG in Archaea: Charging of a UAG-decoding specialized tRNA. Science.

[123] Gaston M.A., Zhang L., Green-Church K.B., Krzycki J.A. (2011). The complete biosynthesis of the genetically encoded amino acid pyrrolysine from lysine. Nature.

[124] Umehara T., Kim J., Lee S., Guo L.T., Söll D., Park H.S. (2012). N-acetyl lysyl-tRNA synthetases evolved by a CcdB-based selection possess N-acetyl lysine specificity in vitro and in vivo. FEBS Lett..

[125] Park H.S., Hohn M.J., Umehara T., Guo L.T., Osborne E.M., Benner J., Noren C.J., Rinehart J., Söll D. (2011). Expanding the genetic code of *Escherichia coli* with phosphoserine. Science.

[126] Zhang M.S., Brunner S.F., Huguenin-Dezot N., Liang A.D., Schmied W.H., Rogerson D.T., Chin J.W. (2017). Biosynthesis and genetic encoding of phosphothreonine through parallel selection and deep sequencing. Nat. Methods.

[127] Moen J.M., Mohler K., Rogulina S., Shi X., Shen H., Rinehart J. (2022). Enhanced access to the human phosphoproteome with genetically encoded phosphothreonine. Nat. Commun..

[128] Hoppmann C., Wong A., Yang B., Li S., Hunter T., Shokat K.M., Wang L. (2017). Site-specific incorporation of phosphotyrosine using an expanded genetic code. Nat. Chem. Biol..

[129] Chin J.W., Santoro S.W., Martin A.B., King D.S., Wang L., Schultz P.G. (2002). Addition of p-azido-L-phenylalanine to the genetic code of *Escherichia coli*. J. Am. Chem. Soc..

[130] Nguyen T.A., Cigler M., Lang K. (2018). Expanding the Genetic Code to Study Protein-Protein Interactions. Angew. Chem. Int. Ed. Engl..

[131] Lee S., Kim J., Koh M. (2022). Recent Advances in Fluorescence Imaging by Genetically Encoded Non-canonical Amino Acids. J. Mol. Biol..

[132] Jones C.M., Robkis D.M., Blizzard R.J., Munari M., Venkatesh Y., Mihaila T.S., Eddins A.J., Mehl R.A., Zagotta W.N., Gordon S.E. (2021). Genetic encoding of a highly photostable, long lifetime fluorescent amino acid for imaging in mammalian cells. Chem. Sci..

[133] Krahn N., Tharp J.M., Crnkovic A., Söll D. (2020). Engineering aminoacyl-tRNA synthetases for use in synthetic biology. Enzymes.

[134] Cho C.C., Blankenship L.R., Ma X., Xu S., Liu W. (2022). The Pyrrolysyl-tRNA Synthetase Activity can be Improved by a P188 Mutation that Stabilizes the Full-Length Enzyme. J. Mol. Biol..

[135] Ambrogelly A., Gundllapalli S., Herring S., Polycarpo C., Frauer C., Söll D. (2007). Pyrrolysine is not hardwired for cotranslational insertion at UAG codons. Proc. Natl. Acad. Sci. USA.

[136] Chen P.R., Groff D., Guo J., Ou W., Cellitti S., Geierstanger B.H., Schultz P.G. (2009). A facile system for encoding unnatural amino acids in mammalian cells. Angew. Chem. Int. Ed. Engl..

[137] Willis J.C.W., Chin J.W. (2018). Mutually orthogonal pyrrolysyl-tRNA synthetase/tRNA pairs. Nat. Chem..

[138] Carla R., Polycarpo S.H., Bérubé A., John L. (2006). Wood, Dieter Söll, Alexandre Ambrogelly Pyrrolysine analogues as substrates for pyrrolysyl-tRNA synthetase. FEBS Lett..

[139] Wright D.E., O’Donoghue P. (2019). The Molecular Architecture of Unnatural Amino Acid Translation Systems. Structure.

[140] Wang Y.S., Fang X., Wallace A.L., Wu B., Liu W.R. (2012). A rationally designed pyrrolysyl-tRNA synthetase mutant with a broad substrate spectrum. J. Am. Chem. Soc..

[141] Yanagisawa T., Umehara T., Sakamoto K., Yokoyama S. (2014). Expanded genetic code technologies for incorporating modified lysine at multiple sites. Chembiochem.

[142] Kavran J.M., Gundllapalli S., O’Donoghue P., Englert M., Söll D., Steitz T.A. (2007). Structure of pyrrolysyl-tRNA synthetase, an archaeal enzyme for genetic code innovation. Proc. Natl. Acad. Sci. USA.

[143] Vargas-Rodriguez O., Sevostyanova A., Söll D., Crnković A. (2018). Upgrading aminoacyl-tRNA synthetases for genetic code expansion. Curr. Opin. Chem. Biol..

[144] Mukai T., Kobayashi T., Hino N., Yanagisawa T., Sakamoto K., Yokoyama S. (2008). Adding l-lysine derivatives to the genetic code of mammalian cells with engineered pyrrolysyl-tRNA synthetases. Biochem. Biophys. Res. Commun..

[145] Gautier A., Nguyen D.P., Lusic H., An W., Deiters A., Chin J.W. (2010). Genetically encoded photocontrol of protein localization in mammalian cells. J. Am. Chem. Soc..

[146] Han S., Yang A., Lee S., Lee H.W., Park C.B., Park H.S. (2017). Expanding the genetic code of Mus musculus. Nat. Commun..

[147] Welegedara A.P., Adams L.A., Huber T., Graham B., Otting G. (2018). Site-Specific Incorporation of Selenocysteine by Genetic Encoding as a Photocaged Unnatural Amino Acid. Bioconjugate Chem..

[148] Fu X., Söll D., Sevostyanova A. (2018). Challenges of site-specific selenocysteine incorporation into proteins by *Escherichia coli*. RNA Biol..

[149] Bröcker M.J., Ho J.M., Church G.M., Söll D., O’Donoghue P. (2014). Recoding the genetic code with selenocysteine. Angew. Chem. Int. Ed. Engl..

[150] Arnér E.S., Sarioglu H., Lottspeich F., Holmgren A., Böck A. (1999). High-level expression in *Escherichia coli* of selenocysteine-containing rat thioredoxin reductase utilizing gene fusions with engineered bacterial-type SECIS elements and co-expression with the selA, selB and selC genes. J. Mol. Biol..

[151] Wright D.E., Altaany Z., Bi Y., Alperstein Z., O’Donoghue P. (2018). Acetylation Regulates Thioredoxin Reductase Oligomerization and Activity. Antioxid. Redox Signal..

[152] Jiang Z., Arnér E.S., Mu Y., Johansson L., Shi J., Zhao S., Liu S., Wang R., Zhang T., Yan G. (2004). Expression of selenocysteine-containing glutathione S-transferase in *Escherichia coli*. Biochem. Biophys. Res. Commun..

[153] Kim H.Y., Gladyshev V.N. (2004). Methionine sulfoxide reduction in mammals: Characterization of methionine-R-sulfoxide reductases. Mol. Biol. Cell.

[154] Cheng Q., Arnér E.S. (2017). Selenocysteine Insertion at a Predefined UAG Codon in a Release Factor 1 (RF1)-depleted *Escherichia coli* Host Strain Bypasses Species Barriers in Recombinant Selenoprotein Translation. J. Biol. Chem..

[155] Thyer R., Robotham S.A., Brodbelt J.S., Ellington A.D. (2015). Evolving tRNA(Sec) for efficient canonical incorporation of selenocysteine. J. Am. Chem. Soc..

[156] Aldag C., Bröcker M.J., Hohn M.J., Prat L., Hammond G., Plummer A., Söll D. (2013). Rewiring translation for elongation factor Tu-dependent selenocysteine incorporation. Angew. Chem. Int. Ed. Engl..

[157] Miller C., Bröcker M.J., Prat L., Ip K., Chirathivat N., Feiock A., Veszpremi M., Söll D. (2015). A synthetic tRNA for EF-Tu mediated selenocysteine incorporation in vivo and in vitro. FEBS Lett..

[158] Lajoie M.J., Rovner A.J., Goodman D.B., Aerni H.R., Haimovich A.D., Kuznetsov G., Mercer J.A., Wang H.H., Carr P.A., Mosberg J.A. (2013). Genomically recoded organisms expand biological functions. Science.

[159] Cheng Q., Roveri A., Cozza G., Bordin L., Rohn I., Schwerdtle T., Kipp A., Ursini F., Maiorino M., Miotto G. (2021). Production and purification of homogenous recombinant human selenoproteins reveals a unique codon skipping event in *E. coli* and GPX4-specific affinity to bromosulfophthalein. Redox Biol..

[160] Baron C., Bock A. (1991). The length of the aminoacyl-acceptor stem of the selenocysteine-specific tRNA(Sec) of Escherichia coli is the determinant for binding to elongation factors SELB or Tu. J. Biol. Chem..

[161] Mukai T., Sevostyanova A., Suzuki T., Fu X., Söll D. (2018). A Facile Method for Producing Selenocysteine-Containing Proteins. Angew. Chem. Int. Ed. Engl..

[162] Fischer N., Neumann P., Bock L.V., Maracci C., Wang Z., Paleskava A., Konevega A.L., Schroder G.F., Grubmuller H., Ficner R. (2016). The pathway to GTPase activation of elongation factor SelB on the ribosome. Nature.

[163] Ohtsuki T., Manabe T., Sisido M. (2005). Multiple incorporation of non-natural amino acids into a single protein using tRNAs with non-standard structures. FEBS Lett..

[164] Krahn N., Fischer J.T., Söll D. (2020). Naturally Occurring tRNAs With Non-canonical Structures. Front Microbiol..

[165] Mukai T., Vargas-Rodriguez O., Englert M., Tripp H.J., Ivanova N.N., Rubin E.M., Kyrpides N.C., Söll D. (2017). Transfer RNAs with novel cloverleaf structures. Nucleic Acids Res..

[166] Evans R.M., Krahn N., Murphy B.J., Lee H., Armstrong F.A., Söll D. (2021). Selective cysteine-to-selenocysteine changes in a [NiFe]-hydrogenase confirm a special position for catalysis and oxygen tolerance. Proc. Natl. Acad. Sc.i USA.

[167] Patel A., Mulder D.W., Söll D., Krahn N. (2022). Harnessing selenocysteine to enhance microbial cell factories for hydrogen production. Front. Catal..

[168] Rakauskaite R., Urbanaviciute G., Ruksenaite A., Liutkeviciute Z., Juskenas R., Masevicius V., Klimasauskas S. (2015). Biosynthetic selenoproteins with genetically-encoded photocaged selenocysteines. Chem. Commun..

[169] Hoffman K.S., Chung C.Z., Mukai T., Krahn N., Jiang H.K., Balasuriya N., O’Donoghue P., Söll D. (2023). Recoding UAG to selenocysteine in *Saccharomyces cerevisiae*. RNA.

[170] Lazard M., Dauplais M., Blanquet S., Plateau P. (2015). Trans-sulfuration Pathway Seleno-amino Acids Are Mediators of Selenomethionine Toxicity in *Saccharomyces cerevisiae*. J. Biol. Chem..

[171] Krishnakumar R., Prat L., Aerni H.R., Ling J., Merryman C., Glass J.I., Rinehart J., Söll D. (2013). Transfer RNA misidentification scrambles sense codon recoding. ChemBioChem.

[172] Wan W., Tharp J.M., Liu W.R. (2014). Pyrrolysyl-tRNA synthetase: An ordinary enzyme but an outstanding genetic code expansion tool. Biochim. Biophys. Acta.

[173] Wan W., Huang Y., Wang Z., Russell W.K., Pai P.J., Russell D.H., Liu W.R. (2010). A facile system for genetic incorporation of two different noncanonical amino acids into one protein in *Escherichia coli*. Angew. Chem. Int. Ed. Engl..

[174] Italia J.S., Addy P.S., Erickson S.B., Peeler J.C., Weerapana E., Chatterjee A. (2019). Mutually Orthogonal Nonsense-Suppression Systems and Conjugation Chemistries for Precise Protein Labeling at up to Three Distinct Sites. J. Am. Chem. Soc..

[175] Tharp J.M., Vargas-Rodriguez O., Schepartz A., Söll D. (2021). Genetic Encoding of Three Distinct Noncanonical Amino Acids Using Reprogrammed Initiator and Nonsense Codons. ACS Chem. Biol..

[176] Italia J.S., Addy P.S., Wrobel C.J., Crawford L.A., Lajoie M.J., Zheng Y., Chatterjee A. (2017). An orthogonalized platform for genetic code expansion in both bacteria and eukaryotes. Nat. Chem. Biol..

[177] Zheng Y., Lajoie M.J., Italia J.S., Chin M.A., Church G.M., Chatterjee A. (2016). Performance of optimized noncanonical amino acid mutagenesis systems in the absence of release factor 1. Mol. BioSystems.

[178] Heinemann I.U., Rovner A.J., Aerni H.R., Rogulina S., Cheng L., Olds W., Fischer J.T., Söll D., Isaacs F.J., Rinehart J. (2012). Enhanced phosphoserine insertion during *Escherichia coli* protein synthesis via partial UAG codon reassignment and release factor 1 deletion. FEBS Lett..

[179] Oba T., Andachi Y., Muto A., Osawa S. (1991). CGG: An unassigned or nonsense codon in Mycoplasma capricolum. Proc. Natl. Acad. Sci. USA.

[180] Schmitt M.A., Biddle W., Fisk J.D. (2018). Mapping the Plasticity of the *Escherichia coli* Genetic Code with Orthogonal Pair-Directed Sense Codon Reassignment. Biochemistry.

[181] Schwark D.G., Schmitt M.A., Fisk J.D. (2021). Directed Evolution of the Methanosarcina barkeri Pyrrolysyl tRNA/aminoacyl tRNA Synthetase Pair for Rapid Evaluation of Sense Codon Reassignment Potential. Int. J. Mol. Sci..

[182] Morosky P., Comyns C., Nunes L.G.A., Chung C.Z., Hoffmann P.R., Söll D., Vargas-Rodriguez O., Krahn N. (2023). Dual incorporation of non-canonical amino acids enables production of post-translationally modified selenoproteins. Front. Mol. Biosci..

[183] Ma X., Karra S., Guo W., Lindner D.J., Hu J., Angell J.E., Hofmann E.R., Reddy S.P., Kalvakolanu D.V. (2001). Regulation of interferon and retinoic acid-induced cell death activation through thioredoxin reductase. J. Biol. Chem..

[184] Anestal K., Arnér E.S. (2003). Rapid induction of cell death by selenium-compromised thioredoxin reductase 1 but not by the fully active enzyme containing selenocysteine. J. Biol. Chem..

[185] Anestal K., Prast-Nielsen S., Cenas N., Arnér E.S. (2008). Cell death by SecTRAPs: Thioredoxin reductase as a prooxidant killer of cells. PLoS ONE.

[186] Zhang Y., Sun S., Xu W., Yang R., Yang Y., Guo J., Ma K., Xu J. (2022). Thioredoxin reductase 1 inhibitor shikonin promotes cell necroptosis via SecTRAPs generation and oxygen-coupled redox cycling. Free Radic. Biol. Med..

[187] Yamauchi I., Sakane Y., Yamashita T., Hakata T., Sugawa T., Fujita H., Okamoto K., Taura D., Hirota K., Ueda Y. (2022). Thyroid hormone economy in mice overexpressing iodothyronine deiodinases. FASEB J..

[188] Weiss N., Zhang Y.Y., Heydrick S., Bierl C., Loscalzo J. (2001). Overexpression of cellular glutathione peroxidase rescues homocyst(e)ine-induced endothelial dysfunction. Proc. Natl. Acad. Sci. USA.

[189] Bierl C., Voetsch B., Jin R.C., Handy D.E., Loscalzo J. (2004). Determinants of human plasma glutathione peroxidase (GPx-3) expression. J. Biol. Chem..

[190] Novoselov S.V., Lobanov A.V., Hua D., Kasaikina M.V., Hatfield D.L., Gladyshev V.N. (2007). A highly efficient form of the selenocysteine insertion sequence element in protozoan parasites and its use in mammalian cells. Proc. Natl. Acad. Sci. USA.

[191] Han X., Fan Z., Yu Y., Liu S., Hao Y., Huo R., Wei J. (2013). Expression and characterization of recombinant human phospholipid hydroperoxide glutathione peroxidase. IUBMB Life.

[192] Yokoo H., Oba M., Uchida S. (2021). Cell-Penetrating Peptides: Emerging Tools for mRNA Delivery. Pharmaceutics.

[193] Kurrikoff K., Vunk B., Langel U. (2021). Status update in the use of cell-penetrating peptides for the delivery of macromolecular therapeutics. Expert Opin. Biol. Ther..

[194] Tian Y., Zhou S. (2021). Advances in cell penetrating peptides and their functionalization of polymeric nanoplatforms for drug delivery. Wiley Interdiscip. Rev. Nanomed. Nanobiotechnol..

[195] Shoari A., Tooyserkani R., Tahmasebi M., Lowik D. (2021). Delivery of Various Cargos into Cancer Cells and Tissues via Cell-Penetrating Peptides: A Review of the Last Decade. Pharmaceutics.

[196] Han K., Jeon M.J., Kim K.A., Park J., Choi S.Y. (2000). Efficient intracellular delivery of GFP by homeodomains of Drosophila Fushi-tarazu and Engrailed proteins. Mol. Cells.

[197] Vives E., Richard J.P., Rispal C., Lebleu B. (2003). TAT peptide internalization: Seeking the mechanism of entry. Curr. Protein Pept. Sci..

[198] Lichtenstein M., Zabit S., Hauser N., Farouz S., Melloul O., Hirbawi J., Lorberboum-Galski H. (2021). TAT for Enzyme/Protein Delivery to Restore or Destroy Cell Activity in Human Diseases. Life.

[199] Kurnaeva M.A., Sheval E.V., Musinova Y.R., Vassetzky Y.S. (2019). Tat basic domain: A “Swiss army knife” of HIV-1 Tat?. Rev. Med. Virol..

[200] Wright D.E., Siddika T., Heinemann I.U., O’Donoghue P. (2022). Delivery of the selenoprotein thioredoxin reductase 1 to mammalian cells. Front. Mol. Biosci..

